# Financial contributions to global surgery: an analysis of 160 international charitable organizations

**DOI:** 10.1186/s40064-016-3046-z

**Published:** 2016-09-13

**Authors:** Lily Gutnik, Gavin Yamey, Robert Riviello, John G. Meara, Anna J. Dare, Mark G. Shrime

**Affiliations:** 1 Department of Surgery, University of Utah, Salt Lake City, UT USA; 2 Center for Surgery and Public Health, Brigham and Women’s Hospital, Boston, MA USA; 3 Tidziwe Center, UNC Project Malawi, Privae Bag A-104, Lilongwe, Malawi; 4 Duke Global Health Institute, Duke University, Durham, NC USA; 5 Department of Surgery, Brigham and Women’s Hospital, Boston, MA USA; 6 Program in Global Surgery and Social Change, Harvard Medical School, Boston, MA USA; 7 Department of Plastic Surgery, Boston Children’s Hospital, Boston, MA USA; 8 King’s Centre for Global Health, King’s Health Partners, King’s College London, London, England, UK; 9 Harvard University Interfaculty Initiative in Health Policy, Boston, MA USA; 10 Office of Global Surgery, Massachusetts Eye and Ear Infirmary, Boston, MA USA; 11 Department of Otology and Laryngology, Harvard Medical School, Boston, MA USA

**Keywords:** Surgery, Global health, Finance, Economics

## Abstract

**Background:**

The non-profit and volunteer sector has made notable contributions to delivering surgical services in low-and middle-income countries (LMICs). As an estimated 55 % of surgical care delivered in some LMICs is via charitable organizations; the financial contributions of this sector provides valuable insight into understanding financing priorities in global surgery.

**Methods:**

Databases of registered charitable organizations in five high-income nations (United States, United Kingdom, Canada, Australia, and New Zealand) were searched to identify organizations committed exclusively to surgery in LMICs and their financial data. For each organization, we categorized the surgical specialty and calculated revenues and expenditures. All foreign currency was converted to U.S. dollars based on historical yearly average conversion rates. All dollars were adjusted for inflation by converting to 2014 U.S. dollars.

**Results:**

One hundred sixty organizations representing 15 specialties were identified. Adjusting for inflation, in 2014 U.S. dollars (US$), total aggregated revenue over the years 2008–2013 was $3·4 billion and total aggregated expenses were $3·1 billion. Twenty-eight ophthalmology organizations accounted for 45 % of revenue and 49 % of expenses. Fifteen cleft lip/palate organizations totaled 26 % of both revenue and expenses. The remaining 117 organizations, representing a variety of specialties, accounted for 29 % of revenue and 25 % of expenses. In comparison, from 2008 to 2013, charitable organizations provided nearly $27 billion for global health, meaning an estimated 11.5 % went towards surgery.

**Conclusion:**

Charitable organizations that exclusively provide surgery in LMICs primarily focus on elective surgeries, which cover many subspecialties, and often fill deep gaps in care. The largest funding flows are directed at ophthalmology, followed by cleft lip and palate surgery. Despite the number of contributing organizations, there is a clear need for improvement and increased transparency in tracking of funds to global surgery via charitable organizations.

## Background

For the past three decades there has been an expanding number of organizations and activity in the non-profit and volunteer sector (Salamon 2010). These sectors account for significant economic contributions to global health and mobilization of thousands of volunteers. Even in the face of the global financial crisis—when levels of overall development assistance for health (DAH) were stagnant, non-governmental organizations (NGOs) increased their spending on DAH by 10 % (2010–2011) (Leach-Kemon et al. 2012; Institute for Health Metrics and Evaluation 2012). Furthermore, surgeons—in conjunction with non-profit and volunteer organizations—have played a prominent role in providing service in low and middle-income countries (LMICs) where access to surgical care remains limited (Casey 2007; Mcqueen et al. 2010; Shrime et al. 2014; Ozgediz 2009; Kingham et al. 2011).

Surgical diseases account for 11–30 % of the global burden of disease, with LMICs bearing largest majority of the burden, despite having the least capacity to manage these conditions (Shrime et al. 2014; Shrime et al. 2015; Debas et al. 2015). Approximately 5 billion people worldwide are without access to timely, affordable, and safe surgery (Alkire et al. 2015).

International charitable organizations providing surgical care in LMICs often work in regions with the highest burden of surgical disease and the least amount of human and financial resources (Mcqueen et al. 2010; Nguyen et al. 2014). Additionally, while these organizations provide a broad range of surgical specialties, the cases, counterintuitively, are mainly elective in nature (Mcqueen et al. 2010). As a result, these organizations mainly adhere to short-term mission models and provide clinical service as well as training opportunities (Nguyen et al. 2014).

Besides delivering needed care, charitable organizations account for significant funding flows to global health. Conservative estimates suggest that total expenditures on short-term medical missions, including surgical missions, are about $250 million per year (Maki et al. 2008). One study found that twenty of the largest U.S. NGOs that have health as a top priority spent $11.8 billion on global health from 2002 to 2006 (Ravishankar et al. 2009). Furthermore, health NGOs as a whole accounted for 15·7 % of the $31·3 billion total sum of DAH in 2013 (Dieleman et al. 2014). Little, however, is known about the finances provided towards surgery in LMICs because DAH databases do not specifically collect data on surgical services.

To this point, there have been no studies dedicated to aggregating the revenue and expenditure of exclusively surgical charitable organizations. As an estimated 55 % of surgical care delivered in some LMICs is via charitable organizations; understanding the financials of the charitable sector wbe helpful in estimating funding flows to global surgery (Bolkan et al. 2015). Furthermore, the dual nature of charitable organizations-as both funding channels and implementation agents- make them a particularly unique topic to study (Dieleman et al. 2014). In this study, we examined financial contributions made to global surgery exclusively by surgical charitable organizations in five high-income nations (United States, United Kingdom, Canada, Australia, and New Zealand).

## Methods

We define charitable organizations as non-profit, non-governmental organizations that serve the public interest. These organizations may receive funding from a variety of sources including private donations, grants, government contracts, and user fees. Charitable organizations with available financial information described here represent the spectrum of platforms for the delivery of surgical care by charitable organizations described by Shrime et al. (2014) short-term surgical trips, specialized hospitals, and self-contained surgical platforms.

Charitable organizations providing exclusively surgical care were identified using the surgical volunteerism listings on the websites of the American College of Surgeons Operation Giving Back, the Society of Pediatric Anesthesiologists, OmniMed, and the U.S. State Department Private Volunteer Organizations registry (American College of Surgeons Operation Giving Back; Private Voluntary Organizations; Omni Med Database of Global Health Service Opportunities; Society for Pediatric Anesthesia Volunteer Medical Services Abroad). Additional searches were performed on the Foundation Center Online Directory, idealist.org, UK Charity Commission, Australia Charity Commission, New Zealand Charity Commission, and Canada Revenue Agency Charity Search. Key words used in these searches are described in Table 1. The website of each organization was then reviewed to ensure it met our inclusion criteria of providing exclusively surgical care, and not other services, in LMICs. Charitable organizations were selected solely based on the location and type of services provided; financial thresholds were not considered. Organizations that soley provided surgical care—not other types of medical care or development aid—were selected to be the study’s primary focus, as groups that provide several varieties of care often, in their accounting, do not disaggregate funds, making identifying surgical allocations nearly impossible.

**Table 1:** Summary of study methods

Country	Organization identification	Sources of financial documents
United States of America	American College of Surgeons Operation Giving Back, the Society of Pediatric Anesthesiologists, OmniMed, and the US State Department Private Volunteer Organizations registry, Foundation Center Online Directory,	Organization’s website, Guidestar, ProPublica, Economics Research Institute (ERI), Citizenaudit.org, National Center for Charitable Statistics at the Urban Institute, and the Foundation Center Online Directory
United Kingdom	UK Charity Commission	Organization’s website, UK Charity Commission
Canada	Canada Revenue Agency Charity	Organization’s website, Canada Revenue Agency Charity
Australia	Australia Charity Commission	Organization’s website, Australia Charity Commission
New Zealand	New Zealand Charity Commission	Organization’s website, New Zealand Charity Commission

Keywords used in search: “global,” “international,” “low resource,” “developing countries/nations” and “surgery”, “obstetrics and gynecology,” “obstetric fistula,” “trauma,” “injury,” “congenital birth defects,” “cleft lip/palate,” “cataract,” “ophthalmology,” “burn,” “reconstructive,” “urology,” “orthopedics,” “club foot,” “neurosurgery,” “hydrocephalus,” “anesthesia,” “hernia,” “cardiac,” and “ENT”

Any non-profit organization that is registered in the United States can qualify for federal tax exemptions. In order to apply for these exemptions, most of these groups are required to file a version of the tax form 990. The 990 forms provide information on the organization’s revenue and expenses and are required to be publically available. The 990 s for each organization used in this study were obtained from the organization’s own website or from other public sources listed in Table 1.

For organizations registered in the UK, Canada, Australia, and New Zealand, annual financial reporting is compiled by the respective country’s national charity commission and is made available on the commission’s website and/or on the individual organization’s website Both websites were searched to obtain the data.

Based on availability, up to five of the most recent annual financial documents were collected for each organization. These five reports encompassed the most recent available years, which were not uniform across the study sample. For example, one organization had available documents for the years 2009–2013, while another organization only had the years 2008–2012 available. Both organizations had 5 years of financial data to review, but not the same 5 years. The data set included a total of 651 financial documents from 160 different organizations. Five documents were available for about 80 % (128) of the organizations. Some organizations had anywhere from 1 to 4 available reports. Specifically, 17 out of the 160 organizations (10 %) included in this study had only one document available. Similarly, 16 (10 %) organizations had four documents available. Each organization was categorized by surgical specialty.

The total revenue and expenditure (broken down further into the categories of program service, management and administration, fundraising, and other) were recorded per organization per year. The expenditure breakdown was not available on every financial document. All foreign currency was converted to U.S. dollars using historical yearly average conversion rates. All nominal dollars were adjusted for inflation by converting to 2014 U.S. dollars using the International Monetary Fund World Economic Outlook database (downloaded April 2014). Data was managed and analyzed in JMP Pro 11 and Microsoft Excel. Ethics approval was not obtained; only public financial data was used and none of the data analyzed was linked to any human subjects or personal private health information.

## Results

One hundred sixty organizations representing 15 different surgical specialties (including anesthesia), were included in the study. 651 documents of financial data ranging from 2007 to 2013 were analyzed. Table 2 analyzes the total revenues and expenses of all organizations, divided by type of surgical specialty, then further divided by the number of organizations identified per specialty. The aggregated total revenue was $3·4 billion. The total expenditure was $3·1 billion. Table 3 delineates expenditure breakdown. Service expense/total expense represents the average percentage of total funds spent on actual program services. The median range is 0.71–1. In other words, on an average aggregated level these organizations spend anywhere from 71 to 100 % of their funding on actual program execution. However, data on expenditure breakdown were very limited, so the range of these figures may not truly reflect reality. Nearly all of these organizations focus on elective surgical procedures.

**Table 2:** Total revenue and expenditure for 160 international charitable organizations 2007–2013 in 2014 U.S. dollars

Type of surgery	Number of organizations	Total revenue (sum)	% of total	Total expenses (sum)	% of total
Ophthalmology	28	$1,556,711,013.71	45.22	$1,516,012,160.15	48.36
Cleft Lip/Palate	15	$912,757,996.82	26.52	$809,238,269.93	25.82
Mix	19	$462,030,763.76	13.42	$478,313,096.97	15.26
Reconstructive	25	$230,820,438.28	6.71	$72,786,337.43	2.32
Cardiac	22	$87,152,341.35	2.53	$82,542,871.03	2.63
Orthopedics	18	$86,650,015.57	2.52	$81,162,974.39	2.59
Pediatric	11	$56,377,801.33	1.64	$49,837,202.80	1.59
Obstetric Fistula	10	$24,953,680.80	0.72	$23,488,194.25	0.75
Neurosurgery	2	$11,273,228.00	0.33	$10,601,253.93	0.34
Urology	1	$4,657,374.00	0.14	$4,191,093.97	0.13
ENT	1	$3,354,510.00	0.10	$566,978.10	0.02
Craniofacial	1	$2,906,726.00	0.08	$3,844,568.83	0.12
General	3	$897,863.21	0.03	$805,652.52	0.03
Anesthesia	1	$817,932.45	0.02	$465,826.27	0.01
Burn	2	$733,727.84	0.02	$657,707.13	0.02
Trauma	1	$59,476.55	0.00	$14,663.53	0.00
All	160	$3,442,154,889.65	100.00	$3,134,528,851.22	100.00

**Table 3:** Breakdown of expenditures for 160 international charitable organizations 2007–2013 in 2014 USD

Type of surgery	Total program service expenses (sum)	% of total	Total management expenses (sum)	% of total	Service expense/total expense (median)
Ophthalmology	$1,146,905,574.00	54.30	$25,232,021.67	27.72	0.904
Cleft Lip/Palate	$501,356,549.10	23.40	$27,124,232.84	29.91	0.782
Mix	$253,328,682.50	11.93	$18,558,637.91	20.37	0.890
Orthopedics	$74,106,734.52	2.65	$2,988,617.99	5.66	0.8528
Cardiac	$59,824,911.09	2.83	$5,198,179.02	5.74	0.858
Pediatric	$38,866,267.74	1.84	$1,852,283.99	4.87	0.838
Reconstructive	$39,263,691.25	1.84	$4,459,333.64	2.05	0.782
Obstetric Fistula	$18,275,700.71	0.87	$1,935,357.54	2.14	0.818
Neurosurgery	$116,048.07	0.01	$11,283.36	0.01	0.885
Urology	$2,944,251.58	0.14	$843,406.30	0.94	0.715
ENT	$460,631.72	0.02	$106,003.33	0.12	0.996
Craniofacial	$3,361,305.62	0.16	$384,823.62	0.42	0.871
Burn	$279,259.59	0.01	$22,984.77	0.03	0.968
General	$236,554.13	0.01	$0.00	0.03	1
All	$2,139,326,162.00	100.00	$88,717,165.99	100.00	

Ophthalmology (n = 28) is the top revenue-generating surgical specialty, generating about $1·5 billion over the studied time frame. Ophthalmology accounted for 45 % of total revenue and 49 % of total expenditure. Cleft/lip palate (n = 15) was the specialty with the second highest revenues ($912 million), generating $912 million and accounting for 26 % of total revenue and 25 % of expenditure. With $462 million, the “mixed” grouping (n = 19)—which includes organizations that do not focus on a single surgical specialty but enlist multidisciplinary surgical teams-had the third largest revenues, accounting for 14 % of total revenue and 16 % of total expenditure. The remaining 15 % of funds are attributed to 98 organizations that represent 12 specialties. Trauma and burn organizations were the least financially supported, although data for these types of organizations was very limited.

## Discussion

The study identified 160 surgical charitable organizations. Between 2007 and 2013, this group generated around $3·4 billion in revenue and spent nearly $3·1 billion. On an annual basis from 2007 to 2013, the organizations collectively generated an average of $573 million. In 2013, charitable organizations providing DAH as a whole generated about $5 billion (Dieleman et al. 2014), which can be extrapolated that in 2013, about 11.5 % of charitable revenue generation ($0.573 billion out of $5 billion) can be accredited to surgical charities.

There are several limitations to this study. First, only charitable organizations that provided surgical care exclusively (i.e. no other service) were included. There are many other charitable organizations that provide significant amount of surgery in addition to many other forms of medical care and overall developmental aid. These organizations were excluded because it is impossible to ascertain from their financial documents precisely what portion of funds are allocated to surgery as opposed to other activities. Nonetheless, it is important to acknowledge that many such organizations exist and make invaluable contributions to global surgery. To illustrate, Medicine Sans Frontiers International had a net income of €1,008,535,702 and net expenditure of €953,000,000 (of which €615,000,000 was on program expenses) in 2013 (MSF 2013). The organization’s financial report includes a very general breakdown of expenditures, with such categories such as personnel, administration, travel and transportation, medical and nutrition, logistics, and several more. An MSF surgical mission would likely span across all these categories, but the organization does not disclose the exact proportions from each that might be attributed to a mission. To further contextualize, in 2013, 77,350 major surgical interventions were performed, but in that same year 2,497,250 measles vaccinations delivered, there were 9,029,100 outpatient consultations, and 341,600 patients were enrolled in HIV care (MSF^2013^). Additionally, independent studies have shown that MSF also provides surgical care in various specialties and among different age groups (Groen et al. 2015; Wong et al. 2015a, b; Alvarado et al. 2015). Even within one of the world’s major medical charitable organization, MSF, surgery has been among the least provided services. Another study searched to identify all charitable organizations providing surgical care in LMICs regardless of other services rendered. They identified 313 organizations, including the 160 analyzed here (Ng-Kamstra et al. 2015).

A second limitation to this study is that only included organizations that had publicly- available data, thus not taking into account the work done and money spent by charitable surgical organizations that don’t disclose data to the public. Perhaps some organizations were not officially registered as a charitable organizations, thus they were not legally required to publically provide financial documents. Therefore, their finances are unknown and could not be included in this study.

A third, related limitation is only organizations that had financial data available in English (we were unable to read reports in other languages). Therefore, many charitable organizations that are registered in the European Union, Asia, Africa, and South America were not included.

Despite these limitations, the countries and organizations included in this study are among the worlds leading contributors to global health. Consequently, while the data aggregated in this study may be an underestimate of the comprehensive amount of money generated and spent for surgery in LMICs, it is representative of the funding flows from charitable organizations in the top donor countries.

A well functioning surgical system is an integral part of any strong health system. Surgical care is required for the treatment of many diseases, across almost all medical disciplines (Rose et al. 2014; Bickler 2015). Unfortunately, it is often beyond the local health system capacity to provide adequate surgical care, leaving a gap that charitable organizations from high-income countries ultimately fill. In certain countries, charitable organizations may be the de facto option for affordable surgical care (Farmer and Kim 2008). Therefore, until health systems of LMICs are strengthened, charitable organizations remain critical to the delivery of surgical care. Thus, understanding these organizational finances will provide some insight to financing flows to surgery in LMICs.

Likewise, charitable organizations are a critical stakeholder as the field of global surgery evolves. This study offers up some evidence that surgical charitable organizations are, at the very least, financially underrepresented, earning just 11.5 % of charitable organization revenue in 2013. While this statistic is striking, it’s difficult to definitively support, which is illustrates another pressing problem. There is an overall lack of financial data on global surgery. Databases that report DAH, such as the Creditor Reporting System (CRS) database, provide detailed information on aid for specific disease categories, such as malaria, but they contain very little information on funds dedicated to global surgery (Technical Guide to terms and data in the Creditor Reporting System (CRS) Aid Activities database). When donors publicly report their DAH in the CRS, they must assign a “purpose code” (i.e. HIV control) to each of the projects that they fund (DAC and CRS code lists). While several of the codes might potentially include surgery—for example, the “medical services” code includes “laboratories, specialized clinics and hospitals (including equipment and supplies)”—the lack of a dedicated code for surgery makes it extremely difficult for researchers to ascertain how much DAH is dedicated to surgical care. Furthermore, with the passing of resolution A 68/31 on Strengthening Emergency and Essential Surgical Care and Anesthesia as a Component of Universal Health Coverage at the 68th World Health Assembly, there will potentially be greater World Health Organization funding towards surgical care in LMIC making the need for robust financial tracking systems of surgery-oriented funds is imperative.

More detailed purpose codes would significantly improve tracking all external financing sources in global health. Additionally, better classification of expenditures would help elucidate exactly how charitable organizations are utilizing their funds, promoting transparency and accountability. It became clear that there is an overall lack of standardized measurement metrics, and in some cases, a complete lack of reporting on what defined a program service expense. Organizations adhering to the short-term mission model best illustrate this point. In this model, surgical teams from high resource settings come to a LMIC for short period of time, often with their own supplies and equipment, and perform a very specific surgery on a group of patients in a defined period of time. While their own time is often voluntary, the cost of their travel (airfare, accommodations, and meals) is often paid for by the organization. If these indirect costs are being counted as, for example, a service expense or administrative expense, it will not be clear from the financial data that these expenses were actually relating to a short-term mission, making it challenging to track funding flows to all elements of charitable surgical care. Consequently, this makes it difficult to fully analyze the allocation of resources and make corresponding recommendations for their most efficient use.

There is a large unmet need of access to even basic, life-saving surgical care in low resource settings (Rose et al. 2015). Moreover, there are significant economic losses in form of country GDP when surgical services are not rendered to those in need (almost 2.5 % GDP by 2030 in LMIC) (Alkire et al. 2015). The cost of scaling up surgical care in LMIC over the years 2012–2030 is estimated at $300-420 billion-4–8 % of annual total health spending in LMICS (Verguet et al. 2015). In order to achieve this goal, the current financial state and funding flows towards surgery in LMIC need to be better elucidated, documented, and disseminated so that national governments, donors, charitable organizations, and any other stakeholders can better plan and allocate funds. Charitable organizations are a particularly crucial stakeholder as they are both a funder and implementer of surgical care. Thus, we urge all organizations to have detailed and transparent accounting practices to clarify current investments and understand funding gaps for future investment and scale up of surgical care.

## References

[CR1] Alkire BC, Raykar NP, Shrime MG (2015). Global access to surgical care: a modelling study. Lancet Glob Health.

[CR2] Alkire BC, Shrime MG, Dare AJ (2015). Global economic consequences of selected surgical diseases: a modelling study. Lancet Glob Health.

[CR3] Alvarado O, Trelles M, Tayler-Smith K (2015). Orthopaedic surgery in natural disaster and conflict settings: how can quality care be ensured?. Int Orthop.

[CR4] American College of Surgeons Operation Giving Back. http://www.operationgivingback.facs.org/. Accessed 30 August 2014

[CR5] Bickler SW, Weiser TG, Kassebaum N, Higashi H, Chang DC, Barendregt JJ, Noormahomed EV, Vos T, Debas HT, Donkor P, Gawande A, Jamison DT, Kruk ME, Mock CN (2015). Global burden of surgical conditions. Essential surgery: disease control priorities.

[CR6] Bolkan HVS, Von Schreeb J, Samai M, Bash-Taqi D, Kamara TB, Salvesen O, Ystgaard B, Wibe A (2015). Met and unmet needs for surgery in Sierra Leone: a comprehensive, retrospective, countrywide survey from all healthcare facilities performing surgeries in 2012. Surgery.

[CR7] Casey KM (2007). The global impact of surgical volunteerism. Surg Clin N Am.

[CR8] DAC and CRS code lists. http://www.oecd.org/dac/stats/dacandcrscodelists.htm. Accessed 29 March 2015

[CR9] Debas HT, Donkor P, Gawande A, Jamison DT, Kruk ME, Mock CN (2015) Disease control priorities, third edition: volume 1. Essential surgery. Washington, DC: World Bank. © World Bank. https://openknowledge.worldbank.org/handle/10986/21568. License: CC BY 3.0 IGO 26740991

[CR10] Dieleman JL, Graves CM, Templin T (2014). Global health development assistance remained steady in 2013 but did not align with recipients’ disease burden. Health Aff.

[CR11] Farmer PE, Kim JY (2008). Surgery and global health: a view from beyond the OR. World J Surg.

[CR12] Groen RS, Trelles M, Caluwaerts S (2015). A cross-sectional study of indications for cesarean deliveries in Medecins Sans Frontieres facilities across 17 countries. Int J Gynaecol Obstet.

[CR13] Institute for Health Metrics and Evaluation (2012). Financing global health 2012: The end of the golden age?.

[CR14] Kingham TP, Price RR, Casey KM (2011). Beyond volunteerism: augmenting surgical care in resource-limited settings. Bull Am Coll Surg.

[CR15] Leach-Kemon K, Chou DP, Schneider MT (2012). The global financial crisis has led to a slowdown in growth of funding to improve health in many developing countries. Health Aff.

[CR16] Maki J, Qualls M, White B (2008). Health impact assessment and short-term medical missions: a methods study to evaluate quality of care. BMC Health Serv Res.

[CR17] Mcqueen KA, Hyder JA, Taira BR (2010). The provision of surgical care by international organizations in developing countries: a preliminary report. World J Surg.

[CR18] MSF (2013) International Financial Report 2013. http://www.msf.org/sites/msf.org/files/international_financial_report_2013.pdf. Accessed 25 May 2015

[CR19] Ng-Kamstra JS, Arya S, Chung TE (2015). Mapping the playing field—a novel web-based strategy to identify non-governmental actors in global surgery. Lancet.

[CR20] Nguyen N, Jacobs JP, Dearani JA (2014). Survey of nongovernmental organizations providing pediatric cardiovascular care in low- and middle-income countries. World J Pediatr Congenit Heart Surg.

[CR21] Omni Med Database of Global Health Service Opportunities. http://www.omnimed.org/Clients/OmniMed/Databasetemplate/Default.aspx. Accessed 28 August 2014

[CR22] Ozgediz D (2009). Voluntarism and the global unmet need for surgery. Arch Surg.

[CR23] Private Voluntary Organizations. http://pvo.usaid.gov/usaid/. Accessed 14 August 2014

[CR24] Ravishankar N, Gubbins P, Cooley RJ (2009). Financing of global health: tracking development assistance for health from 1990 to 2007. Lancet.

[CR25] Rose J, Chang DC, Weiser TG (2014). The role of surgery in global health: analysis of United States inpatient procedure frequency by condition using the Global Burden of Disease 2010 framework. PLoS One.

[CR26] Rose J, Weiser TG, Hider P (2015). Estimated need for surgery worldwide based on prevalence of diseases: a modelling strategy for the WHO Global Health Estimate. Lancet Glob Health.

[CR27] Salamon LM (2010). Putting the civil society sector on the economic map of the world. Ann Public Cooperative Econ.

[CR28] Shrime MG, Sleemi A, Ravilla TD (2014). Charitable platforms in global surgery: a systematic review of their effectiveness, cost-effectiveness, sustainability, and role training. World J Surg.

[CR29] Shrime MG, Bickler SW, Alkire BC, Mock C (2015). Global burden of surgical disease: an estimation from the provider perspective. Lancet Glob Health.

[CR30] Society for Pediatric Anesthesia Volunteer Medical Services Abroad. http://www.pedsanesthesia.org/vmsa_search.iphtml. Accessed 30 August 2014

[CR31] Technical Guide to terms and data in the Creditor Reporting System (CRS) Aid Activities database. http://www.oecd.org/dac/stats/crsguide.htm. Accessed 19 March 2015

[CR32] Verguet S, Alkire BC, Bickler SW (2015). Timing and cost of scaling up surgical services in low-income and middle-income countries from 2012 to 2030: a modelling study. Lancet Glob Health.

[CR33] Wong EG, Dominguez L, Trelles M (2015). Operative trauma in low-resource settings: the experience of Médecins Sans Frontières in environments of conflict, postconflict, and disaster. Surgery.

[CR34] Wong EG, Trelles M, Dominguez L (2015). Operative procedures in the elderly in low-resource settings: a review of medecins sans frontieres facilities. World J Surg.

